# Surface waves magnitude estimation from ionospheric signature of Rayleigh waves measured by Doppler sounder and OTH radar

**DOI:** 10.1038/s41598-018-19305-1

**Published:** 2018-01-24

**Authors:** Giovanni Occhipinti, Florent Aden-Antoniow, Aurélien Bablet, Jean-Philippe Molinie, Thomas Farges

**Affiliations:** 1 Institut de Physique du Globe de Paris, Université Paris Diderot, UMR 7154 Paris, France; 2 Institut Universitaire de France, Paris, France; 3 Office National d’Études et Recherches Aérospatiales, Palaiseau, France; 4 Commissariat à l’Energie Atomique, CEA, DAM, DIF, F-91297 Arpajon, France

## Abstract

Surface waves emitted after large earthquakes are known to induce atmospheric infrasonic waves detectable at ionospheric heights using a variety of techniques, such as high frequency (HF) Doppler, global positioning system (GPS), and recently over-the-horizon (OTH) radar. The HF Doppler and OTH radar are particularly sensitive to the ionospheric signature of Rayleigh waves and are used here to show ionospheric perturbations consistent with the propagation of Rayleigh waves related to 28 and 10 events, with a magnitude larger than 6.2, detected by HF Doppler and OTH radar respectively. A transfer function is introduced to convert the ionospheric measurement into the correspondent ground displacement in order to compare it with classic seismometers. The ground vertical displacement, measured at the ground by seismometers, and measured at the ionospheric altitude by HF Doppler and OTH radar, is used here to compute surface wave magnitude. The ionospheric surface wave magnitude (*M*_*s*_^*iono*^) proposed here introduces a new way to characterize earthquakes observing the signature of surface Rayleigh waves in the ionosphere. This work proves that ionospheric observations are useful seismological data to better cover the Earth and to explore the seismology of the Solar system bodies observing the ionosphere of other planets.

## Introduction

This work has been inspired by the picture of Charles F. Richter at the Seismological Laboratory in Caltech: a visionary Charles with the magnitude equation on the blackboard behind him.

The introduction of seismic magnitude *M*_*L*_ was motivated, first, by the necessity to estimate and compare, locally, the seismic activity in California, mainly recording seismic events with the 7 short-period Wood-Anderson torsion seismometers of the Southern California group^1^. Charles Richter’s intuition that the maximum amplitude in the far-field, 150 km away from the epicenter, was related to seismic surface waves, pushed Gutenberg & Richter^2^ to the forward development of the surface wave magnitude (*M*_*s*_, eq. 1) in order to generalize the magnitude estimation to seismic events measured over the entire Earth at teleseismic distances from the epicenter.

Later analysis and efforts to unify the different magnitudes^3^, particularly for great shallow earthquakes where the divergences from magnitudes become more important, pushed Kanamori^4^ to introduce the moment magnitude *M*_*w*_ directly related to the radiated energy and physical parameters of the rupture (*e.g*., the stress, the source extent, etc.). Additional magnitudes were developed forward, and are currently in use (*e.g*., Kanamori^5^ and references therein). Despite, the unified magnitude is desirable, the relation between the different magnitudes allows a better characterization of the seismic events^5^. The surface wave magnitude *M*_*s*_ clearly highlights the major ground displacement at the teleseismic distance from the epicenter. This displacement is involved in the transfer of energy from the solid part of the planet to the fluid envelopes: nominally the ocean and the atmosphere, as well as the ionized part of the atmosphere, the ionosphere.

1 $${M}_{s}={\mathrm{log}}_{10}(\frac{{d}_{0}}{T})+1.66\,{\mathrm{log}}_{10}({\rm{\Delta }})+3.5$$

After the Alaska earthquake in 1964, the idea that seismic waves, mainly surface Rayleigh waves, were detectable in the atmosphere and ionosphere opens the era of Ionospheric Seismology (see Occhipinti^6^ and references therein). First evidences of the coupling between the solid Earth and the external fluid envelope were observed at the surface/atmosphere interface by barometers^7^, then in the upper atmosphere^8^ and in the ionosphere^9–11^.

Physically, the surface displacement *d*_0_, induced by Rayleigh waves, produces, by dynamic coupling, an acoustic wave that, propagating upward in the atmosphere, is strongly amplified by the combined effects of the decrease of atmospheric density *ρ* and the conservation of kinetic energy *E*_*c*_ = *ρ v*^2^, where *v* is the local velocity perturbed by the wave propagation. Reaching the altitudes over 80 km, the generated acoustic wave interacts with the ionosphere creating strong variations in the plasma velocity and plasma density, detectable by ionospheric sounding (*e.g*., Doppler sounders, OTH radar and also Incoherent Scatter Radar and GPS).

Early measurements of the ionospheric signature of Rayleigh waves, mainly by Doppler sounders, highlighted in the past that Rayleigh waves produce in the atmosphere/ionosphere acoustic waves with frequencies higher than Brunt-Väisälä frequency^12^. Later, this observational hypothesis was supported by normal modes theory applied to a planet with atmosphere^13^. Doppler sounder observations of the ionospheric signature of the Rayleigh waves reproduced the dispersion curve of Rayleigh waves proving that lithosperic proprieties are measurable at the ionospheric altitude^14^. Artru *et al*.^15^ generalized the observations by Doppler sounder for events with magnitude larger than 6.5. Occhipinti *et al*.^16^ extented the detection capability to over-the-horizon (OTH) radars. The new ionospheric seismometers, namely Doppler sounders and OTH radars, showed a plasma oscillation coherent with the propagation of Rayleigh waves until 60 mHz with a comparable noise/signal ratio^6,17^.

**Figure 1:**
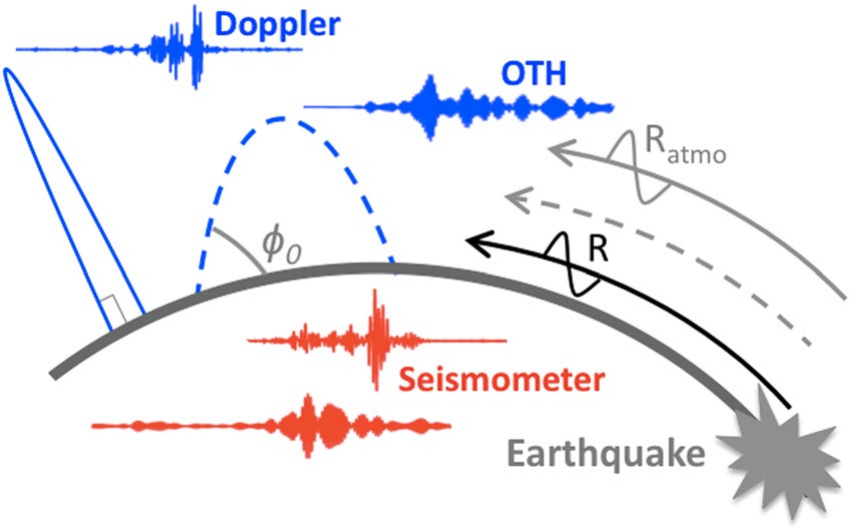
Cartoon of propagation of Rayleigh waves (R, black) and its signature in the atmosphere (R_atmo_, gray) following an Earthquake. The cartoon highlight the observation geometry of the Doppler sounder (blue full-line, emission angle close to 90°) and OTH radar (blue dotted-line, emission angle ϕ_0_, between 10° and 60°). The curves show real measurements of two different events (Colombia, 7.2, 2004-11-15; Philippines, 6.7, 2012-02-06) observed in the ionosphere (blue), by Doppler sounders and OTH respectively, and at the ground (red), by seismometer. The Doppler sounder and the respective seismometer (top) are filtered at around 10–20 mHz, the OTH and the respective seismometer (bottom) are filtered at around 40–50 mHz. The choice of the filtered band is only indicative to highlight that the ionospheric signature of Rayleigh wave is coherent from the Brunt-Väisälä frequency (around 3.5 mHz) and until 60 mHz.

In this work we explore the possibilities to use the signature of Rayleigh waves in the ionosphere (Fig. 1) to estimate the surface wave magnitude (eq. 1^18^) of 38 events: 28 events measured by Doppler sounder and 10 events measured by OTH radar (Tables 1, 2) at teleseismic distance Δ, between 20° and 160°, and recorded at the period *T*, between 15 sec and 300 sec (above the Brunt-Väisälä frequency and until 60 mHz). The Doppler sounder and the OTH radar used in this work are both located in France, for more details about the instruments see Occhipinti *et al*.^16^.

**Table 1:** Seismic events (28) detected by Doppler sounder/Seismometer. Magnitudes *M*_*s*_^*sismo*^ and *M*_*s*_^*iono*^ are calculated at frequency 40–50 mHz.

Event location	date/time	*M*_*s*_^*GCMT*^	*M*_*s*_^*sismo*^	*M*_*s*_^*iono*^	Δ(deg)	Event location	date/time	*M*_*s*_^*GCMT*^	*M*_*s*_^*sismo*^	*M*_*s*_^*iono*^	Δ(deg)
Turkey	1999-08-17 00:01:40	7.8	4.2	Nan	79.44	Costa Rica	1999-08-20 10:02:21	6.9	5.4	6.4	88.99
Taiwan	1999-09-20 17:47:19	7.7	5.8	6.1	152.05	Mexico	1999-09-30 16:31:14	7.5	5.4	6.7	147.08
S. California	1999-10-16 09:46:46	7.4	5.6	6.5	147.08	Turkey	1999-11-12 16:57:21	7.5	4.8	5.7	140.52
New Britain	1999-11-19 13:56:49	7.0	6.2	7.9	63.16	Volcano Islands	2000-03-28 11:00:20	7.6	6.3	6.7	110.60
S. Sumatera	2000-06-04 16:28:27	8.0	7.5	7.1	110.88	Iceland	2000-06-17 15:40:43	6.6	5.4	6.8	99.54
S. Indian Ocean	2000-06-18 14:44:13	7.8	7.4	7.5	80.66	Japan	2000-07-30 12:25:47	6.5	6.6	7.2	72.63
Banda Sea	2000-08-28 15:05:49	6.8	6.0	8.5	144.35	Vanautu Island	2000-10-04 16:58:45	6.9	7.0	8.0	130.25
New Ireland	2000-11-16 07:42:18	7.8	7.9	6.5	144.66	Vanautu Island	2000-01-09 16:49:29	6.6	6.5	6.9	115.93
Kodiak Island	2001-01-10 16:02:43	6.8	5.9	6.7	89.27	El Salvador	2001-01-13 17:33:31	7.9	7.4	6.9	104.38
S. Sumatera	2001-01-16 13:25:09	6.8	5.5	6.5	19.26	Molucca Passage	2001-02-24 07:23:49	7.0	6.3	5.7	100.74
S. Mariana Island	2001-10-12 15:02:18	7.3	7.1	6.8	101.63	China	2001-11-14 09:26:12	8.0	7.3	6.7	129.66
S. Australia	2001-12-12 14:02:37	6.7	5.7	7.4	22.56	Vanuatu Island	2002-01-02 17:22:50	7.5	7.6	5.8	80.07
Papua	2002-09-08 18:44:25	7.8	6.7	Nan	83.63	Loyalty Island	2004-01-03 16:23:20	7.1	7.0	7.8	89.66
Western	2004-09-05 10:07:07	7.0	7.3	6.5	80.22	Colombia	2004-11-15 09:06:56	7.2	7.2	6.3	21.74

**Table 2:** Seismic events (10) detected by OTH radar/Seismometer. Magnitudes *M*_*s*_^*sismo*^ and *M*_*s*_^*iono*^ are calculated at frequency 40–50 mHz.

Event location	date/time	*M*_*s*_^*GCMT*^	*M*_*s*_^*sismo*^	*M*_*s*_^*iono*^	Δ(deg)	Event location	date/time	*M*_*s*_^*GCMT*^	*M*_*s*_^*sismo*^	*M*_*s*_^*iono*^	Δ(deg)
Japan	2011-07-23 04:34:24	6.4	6.3	3.8	71.34	Kermadec	2011-10-21 17:57:16	7.5	7.2	7.9	101.62
Revilla Gigedo	2011-11-01 12:32:01	6.3	5.2	7.7	82.99	Vanuatu Island	2012-02-02 13:34:41	7.1	6.3	7.7	118.10
Philippines	2012-02-06 03:49:13	6.7	6.8	7.0	122.59	Drake Passage	2012-04-14 10:56:19	6.2	5.5	7.6	85.26
Chile	2012-04-17 03:50:16	6.7	6.3	7.6	142.52	Japan	2012-12-07 08:18:23	7.3	6.6	7.6	83.36
Banda Sea	2012-12-10 16:53:09	7.1	5.8	7.7	155.76	Alaska	2013-01-05 08:58:19	7.7	7.5	7.0	80.43

**Figure 2:**
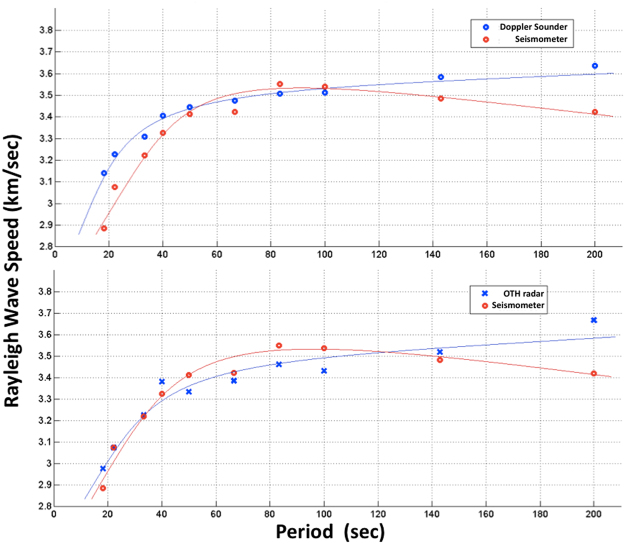
Rayleigh wave dispersion curve computed using 28 seismic events (top) observed by Doppler sounder (blue circle) and seismometer (red circle), and 10 events (bottom) observed by OTH radar (blue cross) and seismometer (red cross).

The clear waveform (Fig. 1) observed in the ionosphere at different frequencies and epicentral distances reproduces perfectly the dispersion curve of the Rayleigh wave (Fig. 2) measured by a single seismometer (Saint Sauveur station, Geoscope network) located in the proximity of the Doppler sounder and OTH radar.

Based on the adiabatic hypothesis of the lower atmosphere, we introduce here a transfer function that allows to transform the ionospheric velocity *v*_*i*_, perturbed by the wave propagation, and measured by Doppler sounder and OTH radar at around 100–300 km of altitude, to the related velocity ${v}_{0}={v}_{i}\sqrt{{\rho }_{i}/{\rho }_{0}}$ at the ground level, where *ρ*_*i*_ and *ρ*_0_ are the density of the neutral atmosphere at the ionospheric altitude *h*_*i*_ and at the ground level respectively. We note that *v*_0_ is the derivate of the ground displacement *d*_0_ during the Rayleigh wave propagation.

The altitude *h*_*i*_ in the ionosphere, where the signal emitted by the Doppler sounder and OTH radar is reflected down (Fig. 1), is estimated using the reflection condition imposed by the Bouguer’s law for the electro-magnetic (EM) waves emitted by the two instruments and propagating into the ionospheric plasma: *r*·*n*·cos*ϕ*, constant along the propagation ray path of the EM wave, where *n* is the refraction i*n*dex, *r* the distance for the Earth center along the ray path, and *ϕ* the local angle between the ray path and the horizon (Fig. 1).

Electromagnetic waves emitted at high frequencies (HF, 3–30 MHz) have the intrinsic property to be reflected/refracted by the ionosphere^19^. The refraction index *n* of the EM wave propagating into the ionospheric plasma at frequency *f*_*e*_ depends on the electron density *N*_*e*_ following eq. 2; where *e* and *m*_*e*_ are the charge and mass of electrons, *ε*_0_ the vacuum permittivity, and *f*_*p*_ the plasma frequency. Doppler sounders and OTH radars usually work taking advantage of this reflection/refraction (Fig. 1).

2 $$n=\sqrt{1-\frac{{N}_{e}{e}^{2}}{4{\pi }^{2}{f}_{e}^{2}{m}_{e}{\varepsilon }_{0}}}=\sqrt{1-\frac{{f}_{p}^{2}}{{f}_{e}^{2}}}$$

Consequently, matching the initial condition at the ground level and at the reflection condition at altitude *h*_*i*_, it is numerically possible to estimate the ionospheric reflection altitude *h*_*i*_ from the Bouguer’s law: ${R}_{\oplus }\cdot \,\cos \,{\varphi }_{0}=({R}_{\oplus }+{h}_{i})\cdot {n}_{i}({h}_{i})$; where *R*_⊕_ is the Earth radius, and *ϕ*_0_ the elevation angle at the emission point at the ground (*ϕ*_0_ = 90° for the Doppler sounder and *ϕ*_0_ = 10°–60° for the OTH radar). We note that the refraction index *n* = 1 at the ground, where *N*_*e*_ = 0.

In order to take into account the daily and seasonal variation of the local density *ρ* in the neutral atmosphere and the electron density *N*_*e*_ in the ionosphere, we use, to estimate the reflection altitude *h*_*i*_ for each single event measured, the specific local atmospheric/ionospheric conditions from the 3D empirical models NRLMSISE-00^20^ for the neutral atmosphere, and the International Reference Ionosphere^21^ for the electron density *N*_*e*_ of the ionospheric plasma, respectively.

Integration of *v*_0_ allows to compute the vertical ground displacement *d*_0_ induced by Rayleigh waves and measured by ionospheric sounding.

This measurement is based on the hypothesis that Doppler sounder and OTH radar sound ionosphere at the altitude where the neutral-plasma coupling is one-to-one, and it not affected yet by the magnetic field, as suggested by Occhipinti *et al*.^22^. Indeed, the effect of the Earth magnetic field, described by the Laurence term of the neutral-plasma coupling equations (*e.g*., eq. 7–9 from Occhipinti *et al*.^22^), is amplified by the plasma density background, consequently the magnetic filed effect is notable mainly at the maximum of ionization (at around 300 km of altitude) above the typical reflection altitude of Doppler sounder and OTH radar (Figs S7–12).

The measurement or the estimation of the ground displacement *d*_0_, by seismometers at the ground, or by Doppler sounder and OTH radar in the ionosphere, allows to compute the surface wave magnitude following eq. 1. We highlight that in seismology the surface wave magnitude is usually computed measuring surface Rayleigh waves at around 20 sec (50 mHz). We call here *M*_*s*_^*seismo*^ and *M*_*s*_^*iono*^ the surface wave magnitude calculated from ground and ionospheric measurements respectively, with a single seismometer or a single ionospheric measurement (by Doppler sounder or OTH radar). We also note that all the events used in this work have magnitude smaller or equal to 8, consequently the surface wave magnitude estimation is not affected by the magnitude saturation^23^. Anyway, for larger events, magnitude saturation affects in the same way seismic and ionospheric measurements.

**Figure 3:**
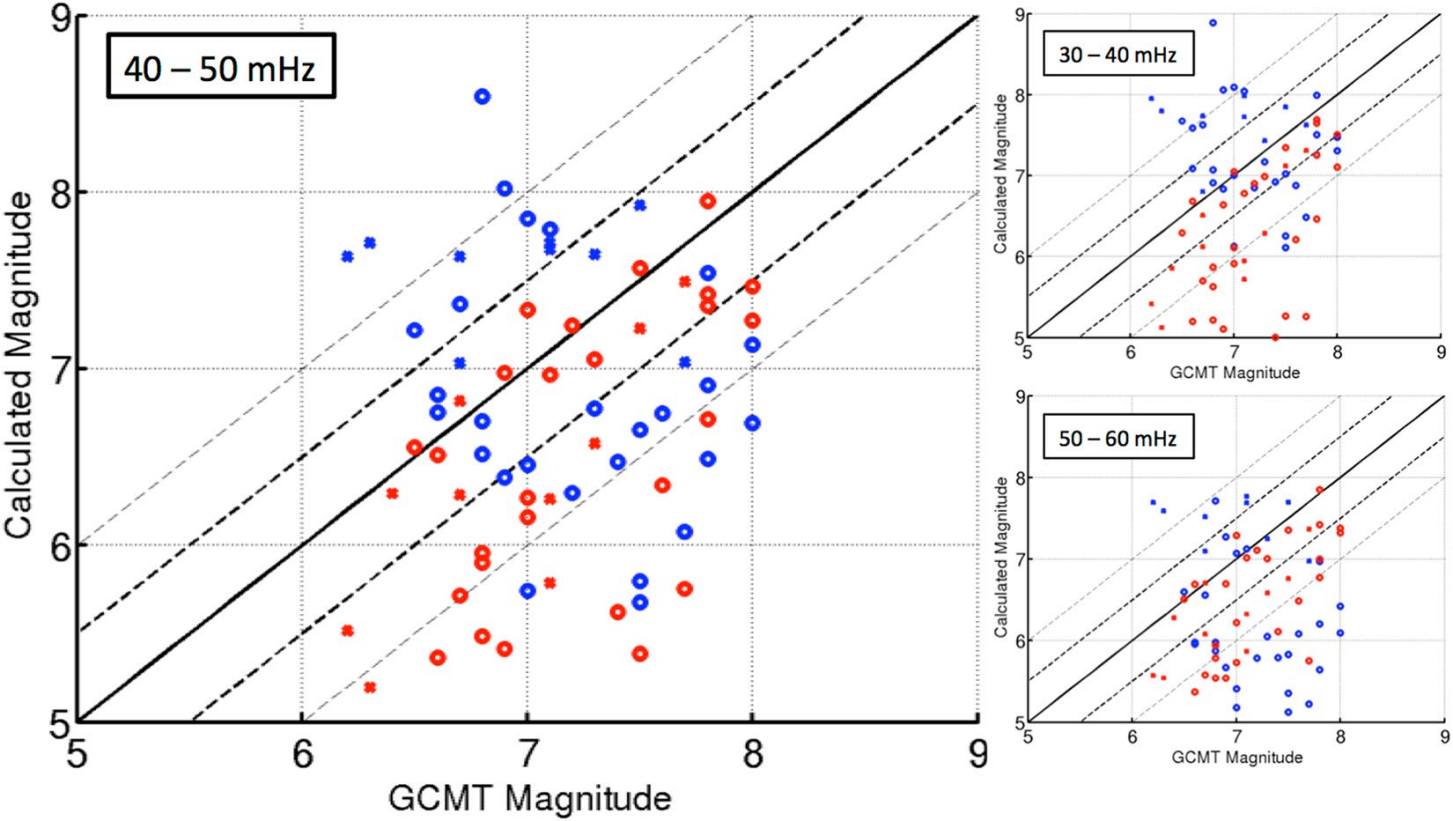
Discrepancies between the official surface wave magnitude estimated by the GCMT and the surface wave magnitude measured with a single seismometer (red cross and red circle), Doppler sounder (blue circle) and OTH radar (blue cross). The different plots show different frequency range used to filter the data for the magnitude computation. Doppler sounder/seismometer and OTH radar/seismometer are measuring 34 and 20 seismic events respectively (Tables 1, 2). Generally, blue show measurement in the ionosphere (by Doppler sounder and OTH radar) and red at the ground level by a single seismometer (Saint Sauveur station, Geoscope network).

Comparison between *M*_*s*_^*seismo*^ and *M*_*s*_^*iono*^ with a reference surface wave magnitude *M*_*s*_^*GCMT*^ (from Global Centroid Moment Tensor, CMT, http://www.globalcmt.org), clearly shows that the sensitivity of a single ionospheric measurement is comparable with a single seismometer (Fig. 3), particularly at frequency between 30 mHz and 60 mHz.

**Figure 4:**
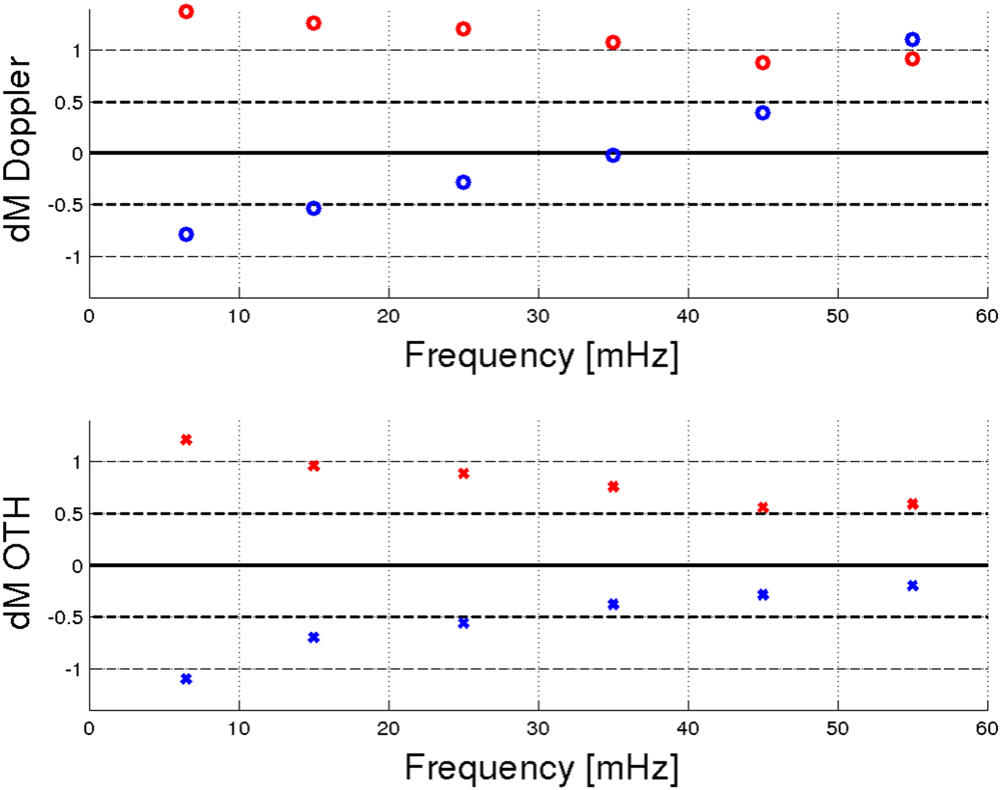
Frequency dependence of the mean value of the discrepancies between the official surface wave magnitude estimated by the GCMT and the surface wave magnitude measured with a single seismometer (red cross and circle), Doppler sounder (blue circle) and OTH radar (blue cross) showed in Fig. 3.

Surprising, between 30 mHz and 40 mHz the mean discrepancy *dM* from the reference magnitude *M*_*s*_^*GCMT*^ for the 28 events detected by Doppler sounder is close to zero, showing that *M*_*s*_^*iono*^ estimated by Doppler sounder perfectly matches the reference magnitude *M*_*s*_^*GCMT*^. More generally, at the frequency between 30 mHz and 60 mHz the mean discrepancy *dM* is always smaller for the Doppler sounder and OTH radar than for the seismometer (Fig. 4), proving that the ionospheric sounding is a valuable and rich seismological observable technique.

The mean discrepancy *dM* becomes larger at smaller frequency, but *M*_*s*_^*iono*^ estimated by both, Doppler sounder and OTH radar, is always closer to the *M*_*s*_^*GCMT*^ than the *M*_*s*_^*seismo*^ estimated by the seismometer.

We note a systematic overestimation of the *M*_*s*_^*seismo*^ and underestimation of *M*_*s*_^*iono*^ compared to the reference magnitude *M*_*s*_^*GCMT*^, particularly at low frequency. This effect becomes less evident at higher frequency (above 40 mHz), and, in particular for the Doppler sounder, the estimated *M*_*s*_^*iono*^ matches perfectly the *M*_*s*_^*seismo*^ from a single seismometer. This effect is related to the limit of the atmospheric and ionospheric models that strongly affect the transfer function and could be reduced and better understood using global scale observations with several ionospheric sounding networks.

Anyway, the mean discrepancy *dM* estimated with a single ionospheric measurement is coherent with the error of 1.5 magnitude-units generally observed using a single seismometer^24^.

We additionally explored the possible link between the ionospheric magnitude estimation and the ionospheric weather conditions (daily time, month, solar flux; S1–S6), as well as the seismic event characteristics (depth, epicentral distance; S7–S12) without any evidence of dependence. Additionally, no-relation between the mean discrepancy *dM* and the reflection altitude (see S7–S12) strongly support the hypothesis of one-to-one coupling between the neutral and plasma.

This surprising result opens terrific perspective in seismology: first of all, the ionospheric measurement by Doppler sounder and OTH radar could be included in the seismic database as GPS has been included in the last decade^25–28^ performing a better coverage of the planet. Today, the Doppler sounders and OTH radars count several permanent and active stations respectively. Additionally, OTH radars are able to sound ionospheric points far away from the radar and usually covering oceanic zones with poor seismic station coverage. In addition to the French OTH radar Nostradamus^29^ that we used here, and that covers partially the Mediterranean sea and the North Atlantic ocean (Europe coast), we count several notable OTH radars: *e.g*., The Jindalee Operational Radar Network (JORN) in Australia, that broadly covers the ocean between Sumatra, Java, Banda see and until to Salomon Islands, all zones with an extremely intense seismic activity; the 6 OTH-Backscattered (OTH-B) US radars located in the East and West coasts, and active until 2007 -cold-storage today-, that covers a large part of North Atlantic and Pacific oceans; the American Relocatable OTH radar (ROTH-R) -today used to monitor the illegal drug trade- that covers Central America and Caribbean, also interesting seismic oceanic zones. Similar seismic measurement of the Rayleigh wave signature in the ionosphere, has been already performed by the Super Dual Auroral Radar Network (SuperDARN) for the case of the Tohoku event in 2011^30^; the SuperDARN entirely cover the North and South poles with 35 radars opening terrific perspective in arctic seismology.

Secondly, the measurement of the seismic surface waves signature in the ionosphere allows to estimate the part of the energy transferred in the fluid envelopes and gives additional information about the coupling phenomena between the solid Earth and the atmosphere/ionosphere.

Finally, the measurement of the Rayleigh waves and magnitude estimation *M*_*s*_^*iono*^ from the ionospheric observations could open futuristic perspectives in planetology allowing to measure seismic activity in other planets by remote atmospheric/ionospheric sounding, *e.g*., on Venus, solving the tricky problem of landing and surviving of a seismometer in hostile environment. Indeed, all the landing mission on Venus, *e.g*., the Russian Venera landers, survived less than 2 hours, making impossible any seismic measurement perspectives and consequent accurate knowledge of the internal structure of the planet.

Introducing the ionospheric magnitude *M*_*s*_^*iono*^ we wish to improve the seismic coverage on the Earth and extend the magnitude estimation to the entire Solar system and beyond.

## Electronic supplementary material

Supplementary Materials
