# Supplementary material for: Surface waves magnitude estimation from ionospheric signature of Rayleigh waves measured by Doppler sounder and OTH radar
Source: Sci Rep. 2018 Jan 24;8:1555. doi: 10.1038/s41598-018-19305-1 (PMC5784152; doi:10.1038/s41598-018-19305-1)
Supplement: Supplementary file 1 — Supplementary Materials [file 41598_2018_19305_MOESM1_ESM.pdf]

Supplementary Materials for

Title: Surface waves magnitude estimation from ionospheric signature of Rayleigh waves measured by Doppler sounder and OTH radar

Authors: Giovanni Occhipinti[1,2,*], Florent Aden-Antoniow[1], Aurélien Bablet[1], Jean-Philippe Molinie[3], Thomas Farges[4].

Affiliations:
[1]Institut de Physique du Globe de Paris, Université Paris Diderot, UMR 7154, France.
[2]Institut Universitaire de France, France.
[3]Office National d'Études et Recherches Aérospatiales, France.
[4]Commissariat à l'Energie Atomique, CEA, DAM, DIF, F-91297 Arpajon, France.
*Corresponding author: ninto@ipgp.fr.

correspondence to: ninto@ipgp.fr

This PDF file includes:

Figs. S1 to S12

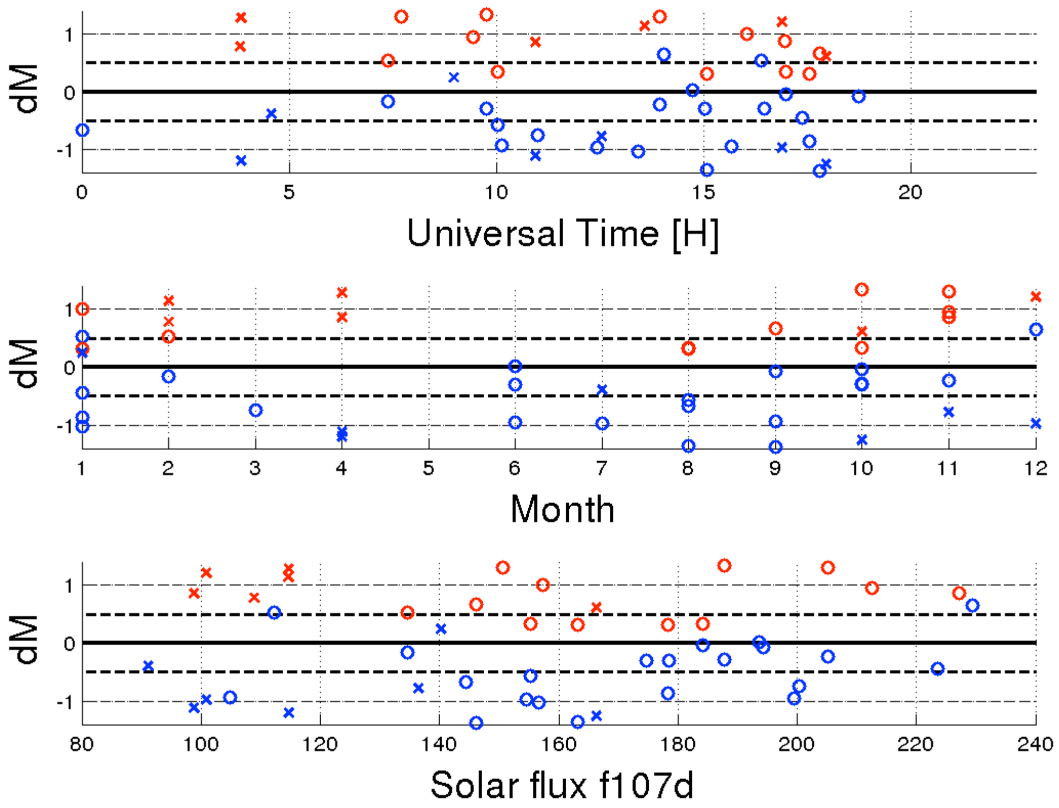

Fig. S1. Daily time, month, solar flux dependence of the mean value of the discrepancies between the official surface wave magnitude estimated by the GCMT and the surface wave magnitude measured with a single seismometer (red cross and circle), Doppler sounder (blue circle) and OTH radar (blue cross). Results obtained at frequency range 3-10 mHz.

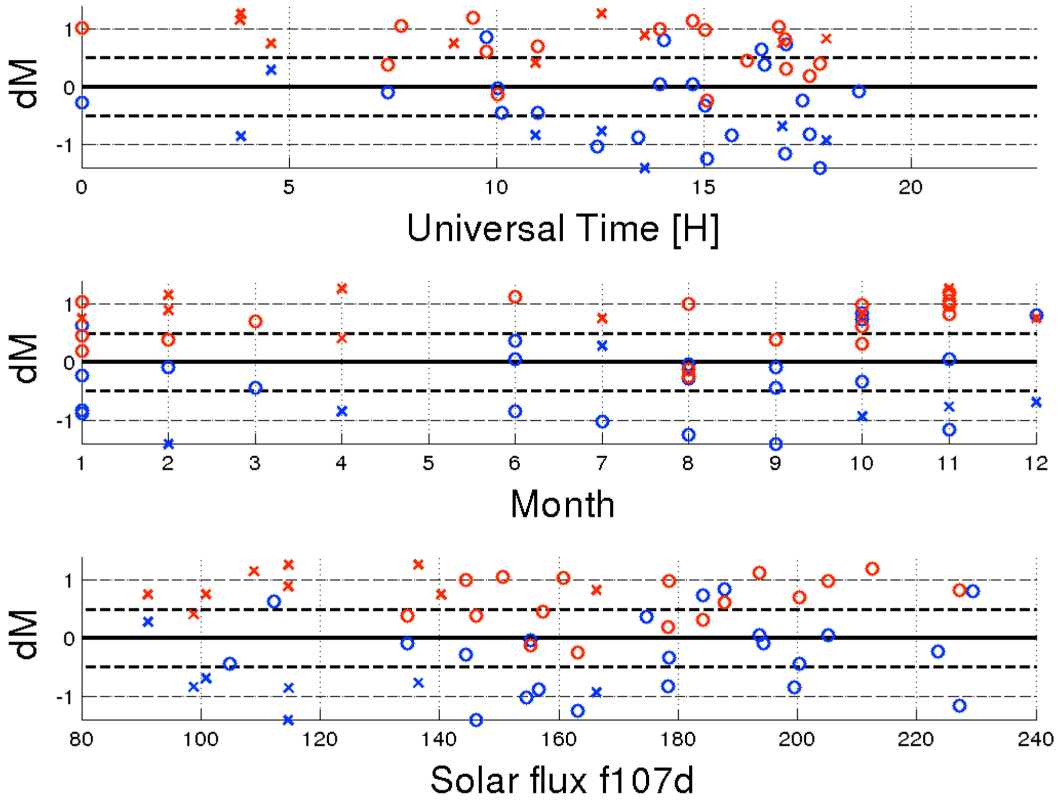

Fig. S2. Daily time, month, solar flux dependence of the mean value of the discrepancies between the official surface wave magnitude estimated by the GCMT and the surface wave magnitude measured with a single seismometer (red cross and circle), Doppler sounder (blue circle) and OTH radar (blue cross). Results obtained at frequency range 10-20 mHz.

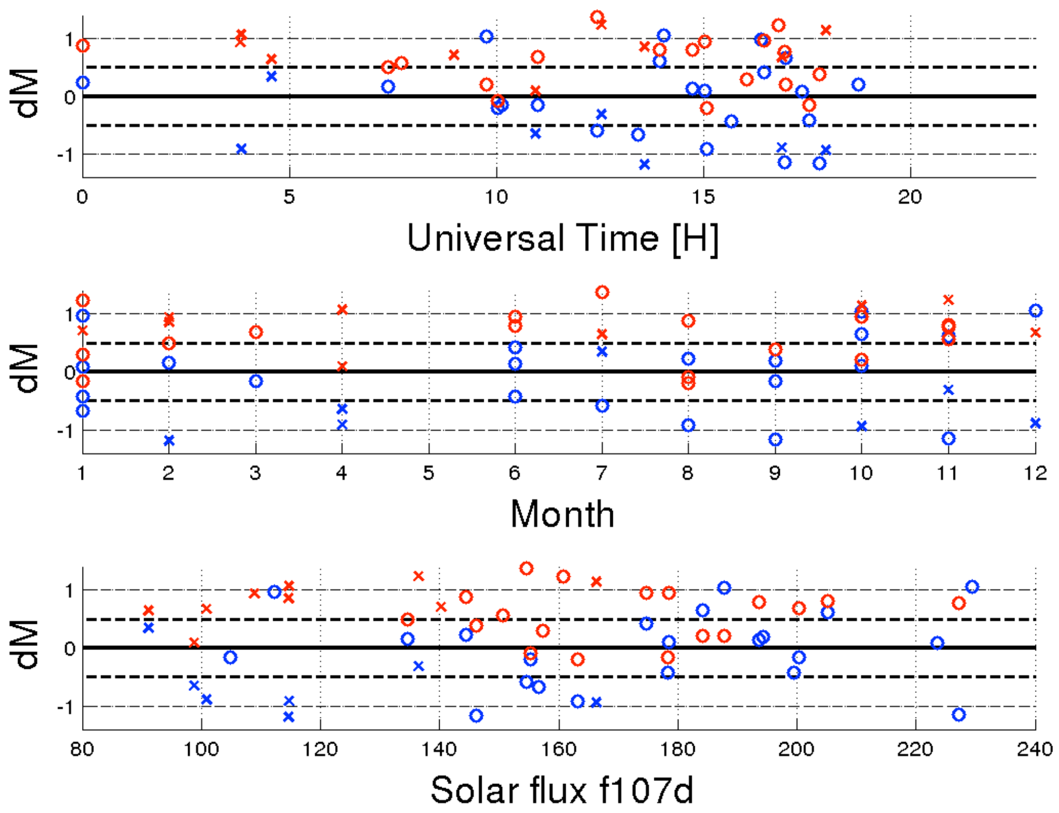

Fig. S3. Daily time, month, solar flux dependence of the mean value of the discrepancies between the official surface wave magnitude estimated by the GCMT and the surface wave magnitude measured with a single seismometer (red cross and circle), Doppler sounder (blue circle) and OTH radar (blue cross). Results obtained at frequency range 20-30 mHz.

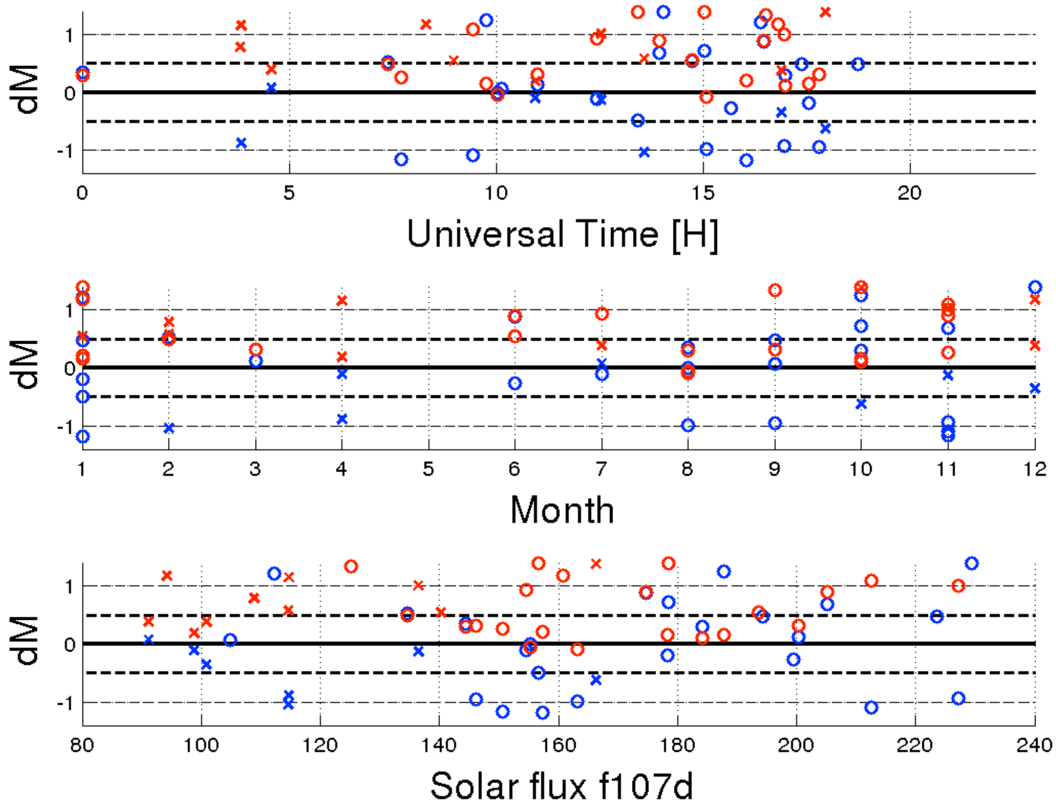

Fig. S4. Daily time, month, solar flux dependence of the mean value of the discrepancies between the official surface wave magnitude estimated by the GCMT and the surface wave magnitude measured with a single seismometer (red cross and circle), Doppler sounder (blue circle) and OTH radar (blue cross). Results obtained at frequency range 30-40 mHz.

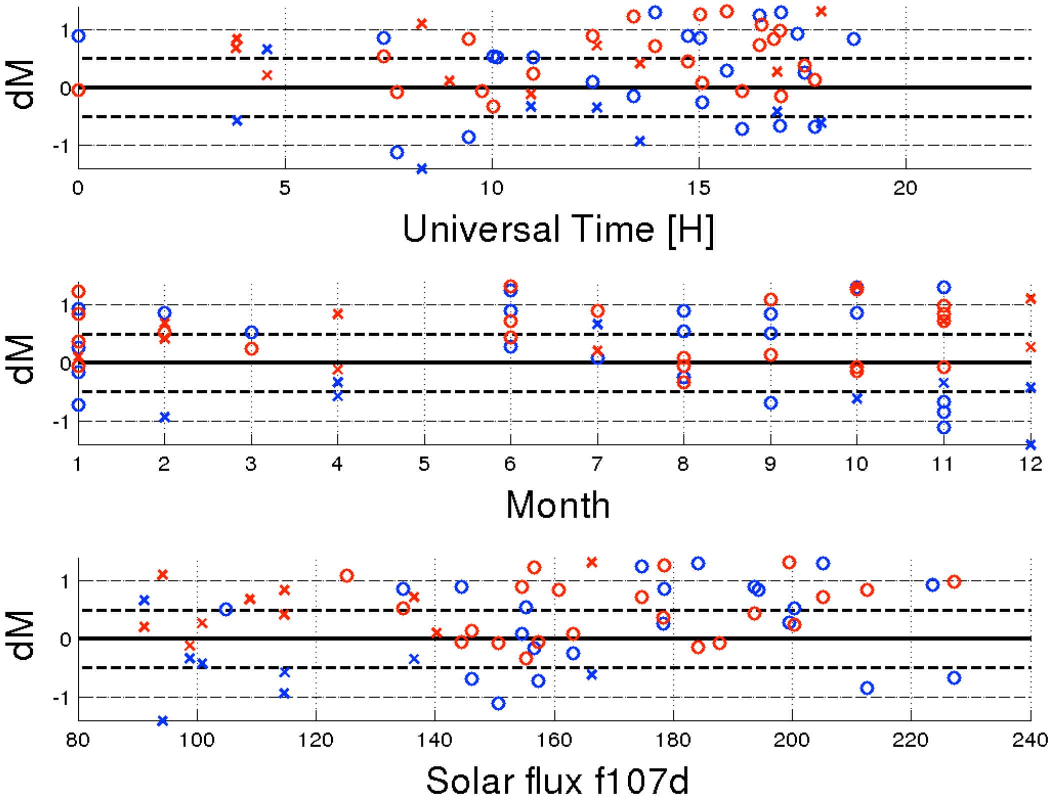

Fig. S5. Daily time, month, solar flux dependence of the mean value of the discrepancies between the official surface wave magnitude estimated by the GCMT and the surface wave magnitude measured with a single seismometer (red cross and circle), Doppler sounder (blue circle) and OTH radar (blue cross). Results obtained at frequency range 40-50 mHz.

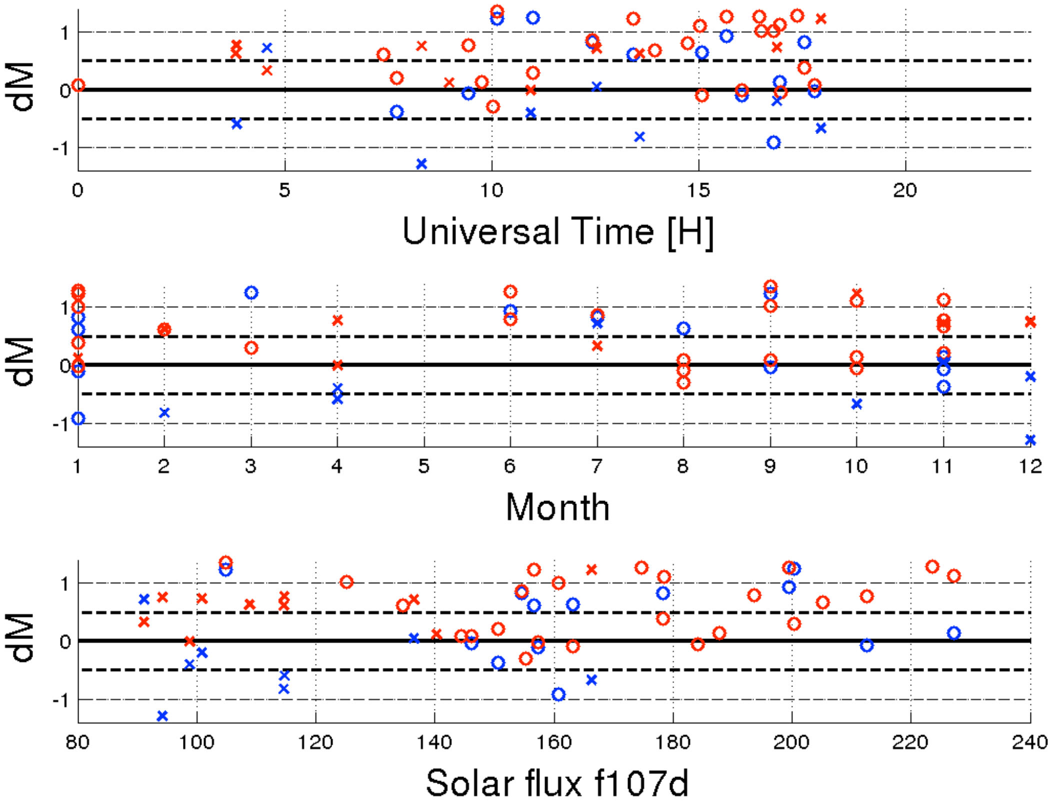

Fig. S6. Daily time, month, solar flux dependence of the mean value of the discrepancies between the official surface wave magnitude estimated by the GCMT and the surface wave magnitude measured with a single seismometer (red cross and circle), Doppler sounder (blue circle) and OTH radar (blue cross). Results obtained at frequency range 50-60 mHz.

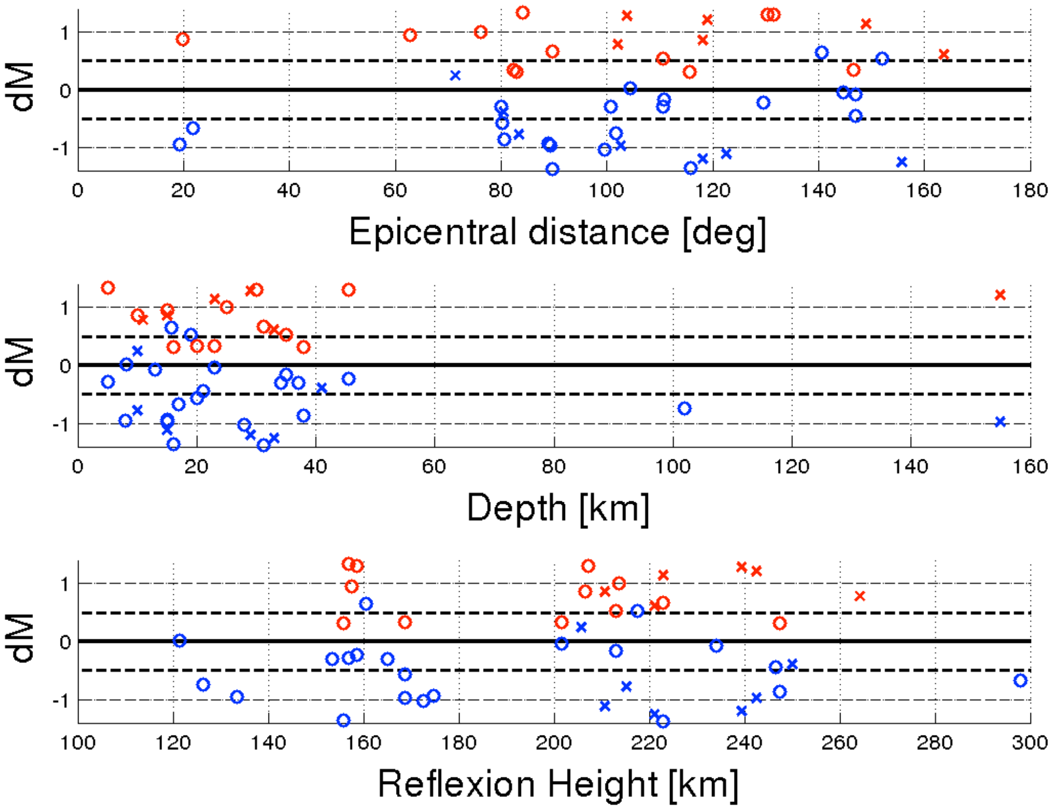

Fig. S7. Epicentral distance, depth, reflexion height dependence of the mean value of the discrepancies between the official surface wave magnitude estimated by the GCMT and the surface wave magnitude measured with a single seismometer (red cross and circle), Doppler sounder (blue circle) and OTH radar (blue cross). Results obtained at frequency range 3-10 mHz.

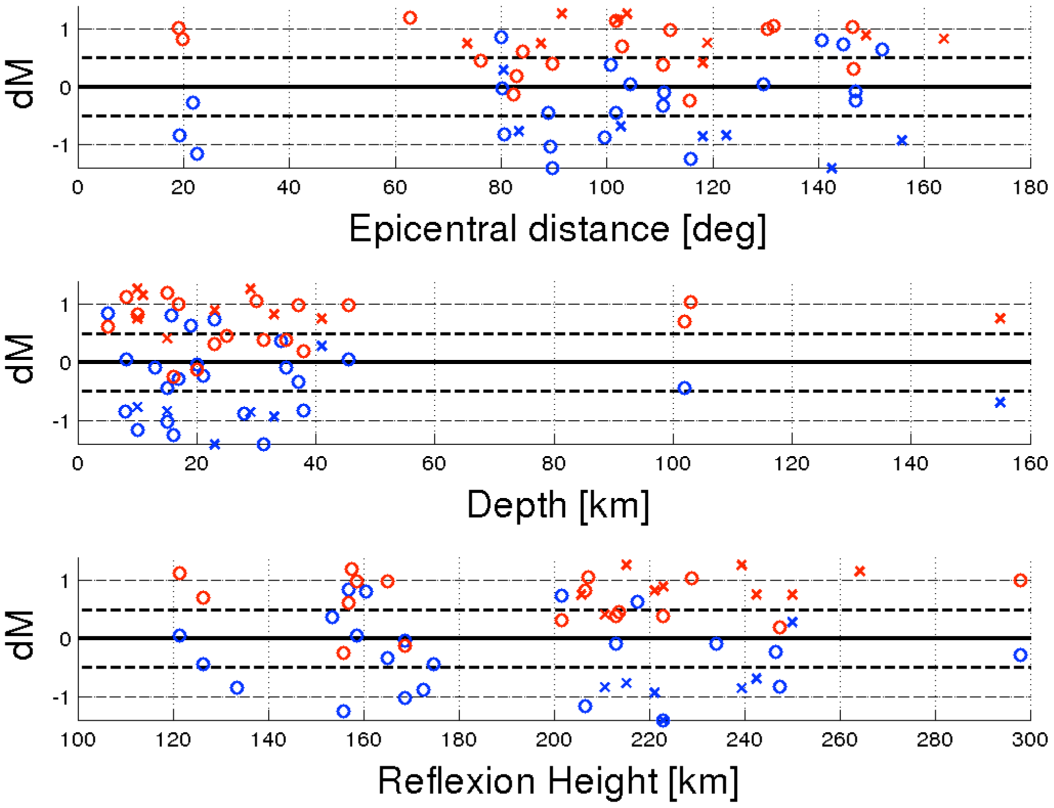

Fig. S8. Epicentral distance, depth, reflexion height dependence of the mean value of the discrepancies between the official surface wave magnitude estimated by the GCMT and the surface wave magnitude measured with a single seismometer (red cross and circle), Doppler sounder (blue circle) and OTH radar (blue cross). Results obtained at frequency range 10-20 mHz.

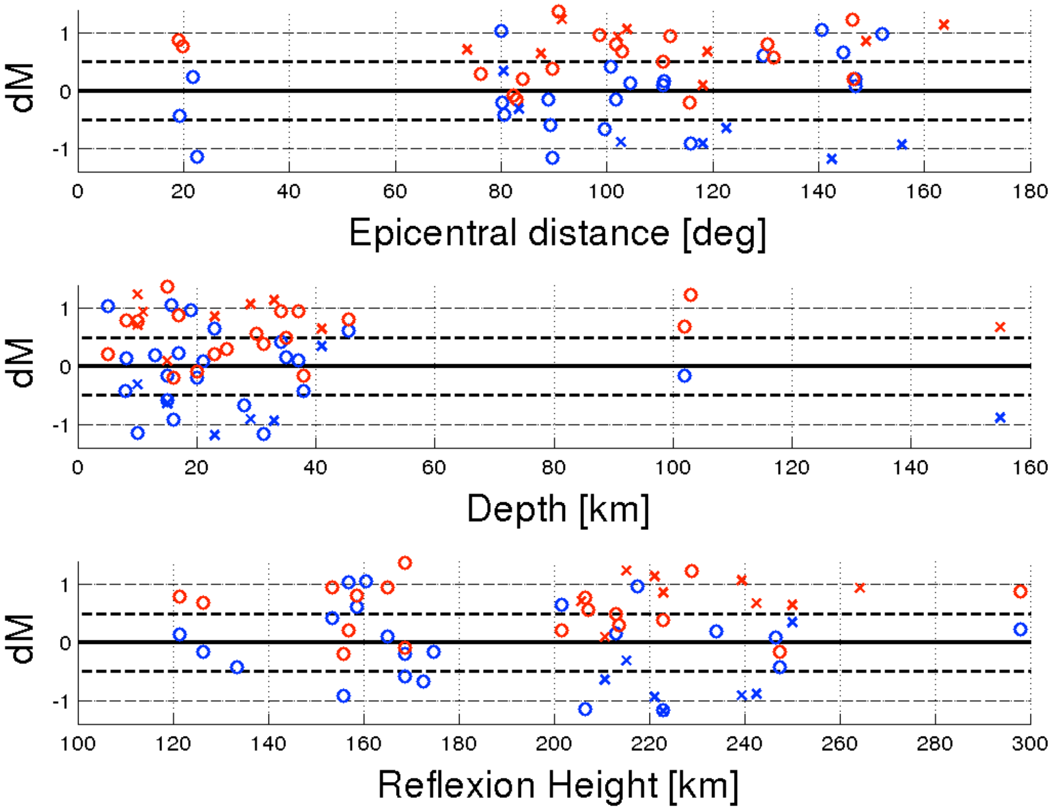

Fig. S9. Epicentral distance, depth, reflexion height dependence of the mean value of the discrepancies between the official surface wave magnitude estimated by the GCMT and the surface wave magnitude measured with a single seismometer (red cross and circle), Doppler sounder (blue circle) and OTH radar (blue cross). Results obtained at frequency range 20-30 mHz.

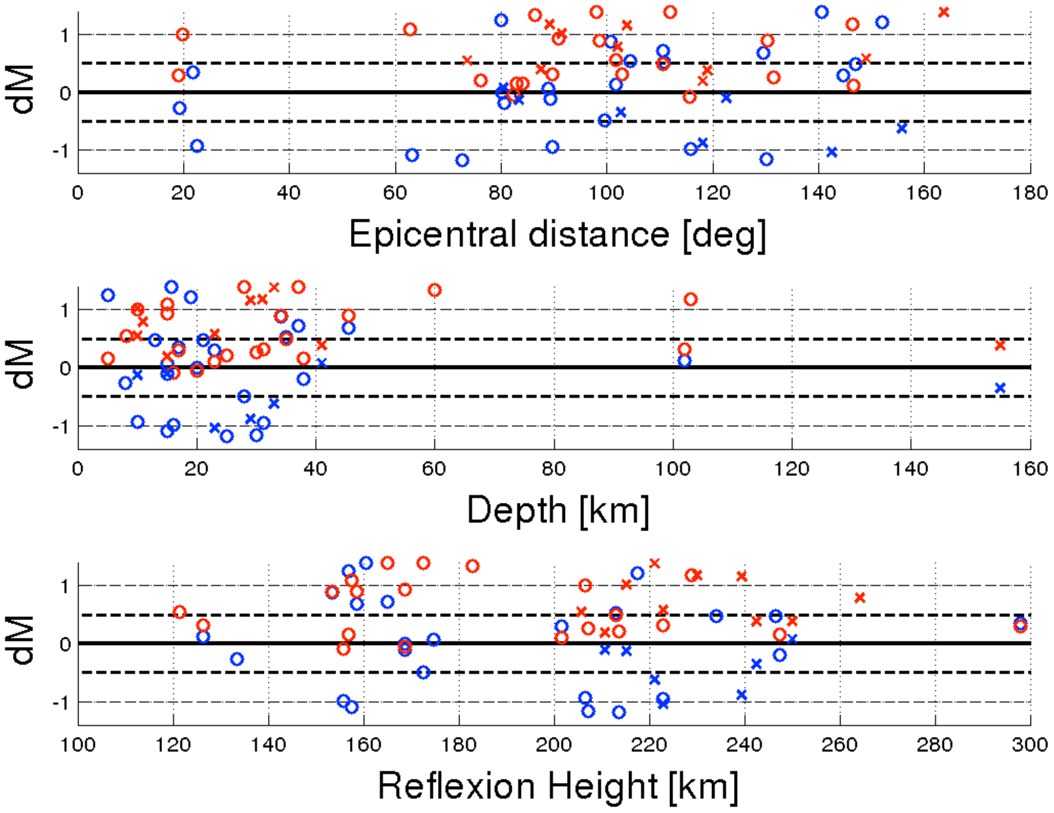

Fig. S10. Epicentral distance, depth, reflexion height dependence of the mean value of the discrepancies between the official surface wave magnitude estimated by the GCMT and the surface wave magnitude measured with a single seismometer (red cross and circle), Doppler sounder (blue circle) and OTH radar (blue cross). Results obtained at frequency range 30-40 mHz.

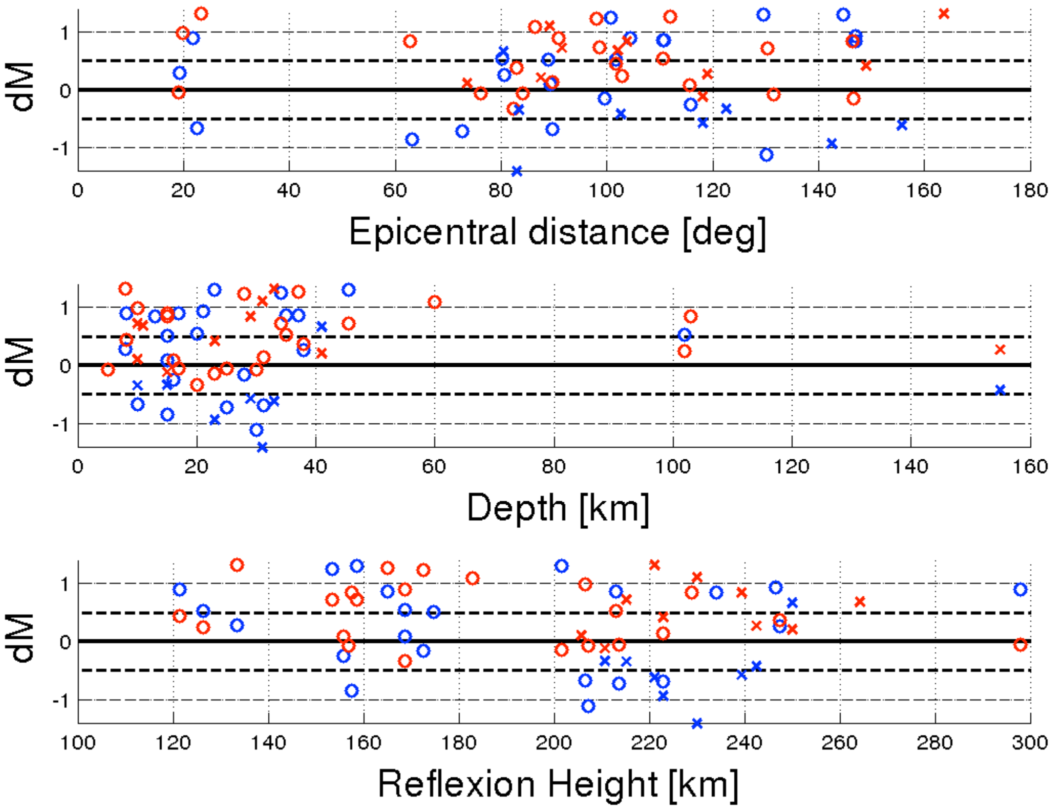

Fig. S11. Epicentral distance, depth, reflexion height dependence of the mean value of the discrepancies between the official surface wave magnitude estimated by the GCMT and the surface wave magnitude measured with a single seismometer (red cross and circle), Doppler sounder (blue circle) and OTH radar (blue cross). Results obtained at frequency range 40-50 mHz.

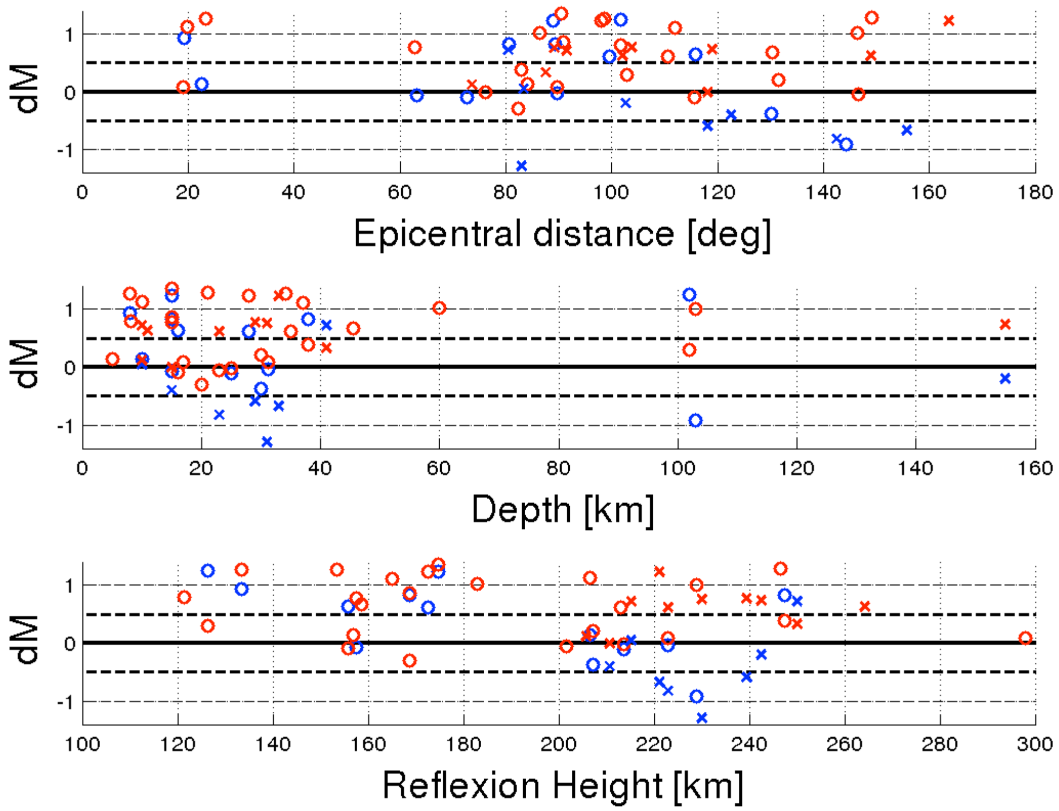

Fig. S12. Epicentral distance, depth, reflexion height dependence of the mean value of the discrepancies between the official surface wave magnitude estimated by the GCMT and the surface wave magnitude measured with a single seismometer (red cross and circle), Doppler sounder (blue circle) and OTH radar (blue cross). Results obtained at frequency range 50-60 mHz.
